# Supplementary material for: The Novel Effector Ue943 Is Essential for Host Plant Colonization by Ustilago esculenta
Source: J Fungi (Basel). 2023 May 19;9(5):593. doi: 10.3390/jof9050593 (PMC10219421; doi:10.3390/jof9050593)
Supplement: Supplementary file 1 [file jof-09-00593-s001.zip › Suppment data/Figure S1.docx]

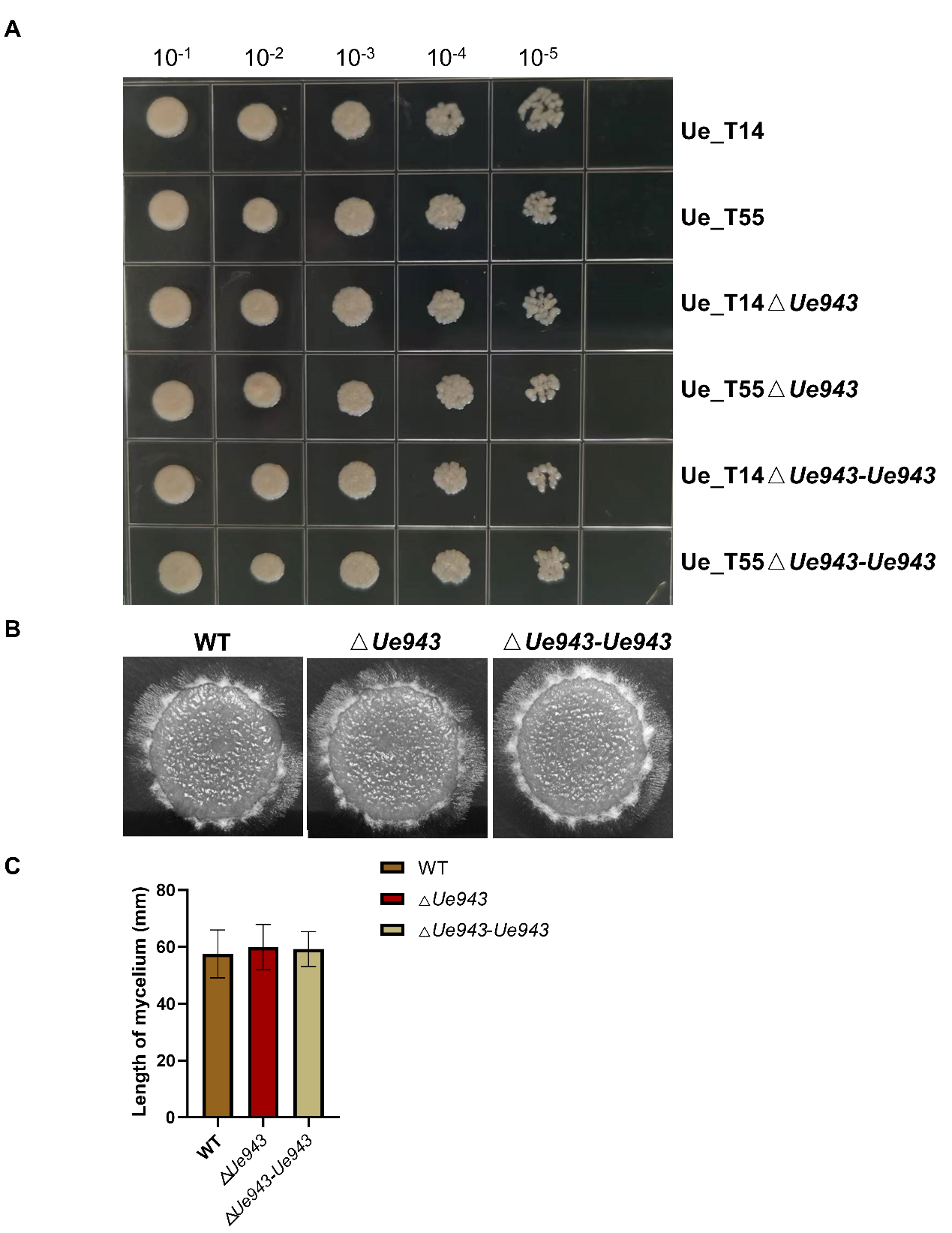


Figure S1. Effects of *Ue943* deletion complementation strains (*ΔUe943-Ue943*) on haploid growth and mating. Δ*Ue943-Ue943* did not effect on haploid growth and mating. They were grown on YEPS medium. WT (UeT14 and UeT55 cross). *ΔUe943* (UeT14*ΔUe943* and UeT55*ΔUe943* cross). *ΔUe943-Ue943 (*UeT14*ΔUe943-Ue943* and UeT55*ΔUe943-Ue943* cross). (A) *Ue943* deletion complementation strains didn't impair haploid growth. Photograph the 72 h haploid growth state with camera. (B) Compared with WT strains and *Ue943* deletion strains. *Ue943* deletion complementation strains didn't impair hypha mating. Photographs were taken at 72 h under a stereo microscope. (C) Mycelia length around colonies of WT, Δ*Ue943*, Δ*Ue943-Ue943 U. esculenta* were monitored at 72 hours incubation on YEPS solid medium (Mean ± SD, n=8). The mycelia length was analyzed with the ImageJ software.
