# Supplementary material for: The Novel Effector Ue943 Is Essential for Host Plant Colonization by Ustilago esculenta
Source: J Fungi (Basel). 2023 May 19;9(5):593. doi: 10.3390/jof9050593 (PMC10219421; doi:10.3390/jof9050593)
Supplement: Supplementary file 1 [file jof-09-00593-s001.zip › Suppment data/Figure S3.docx]

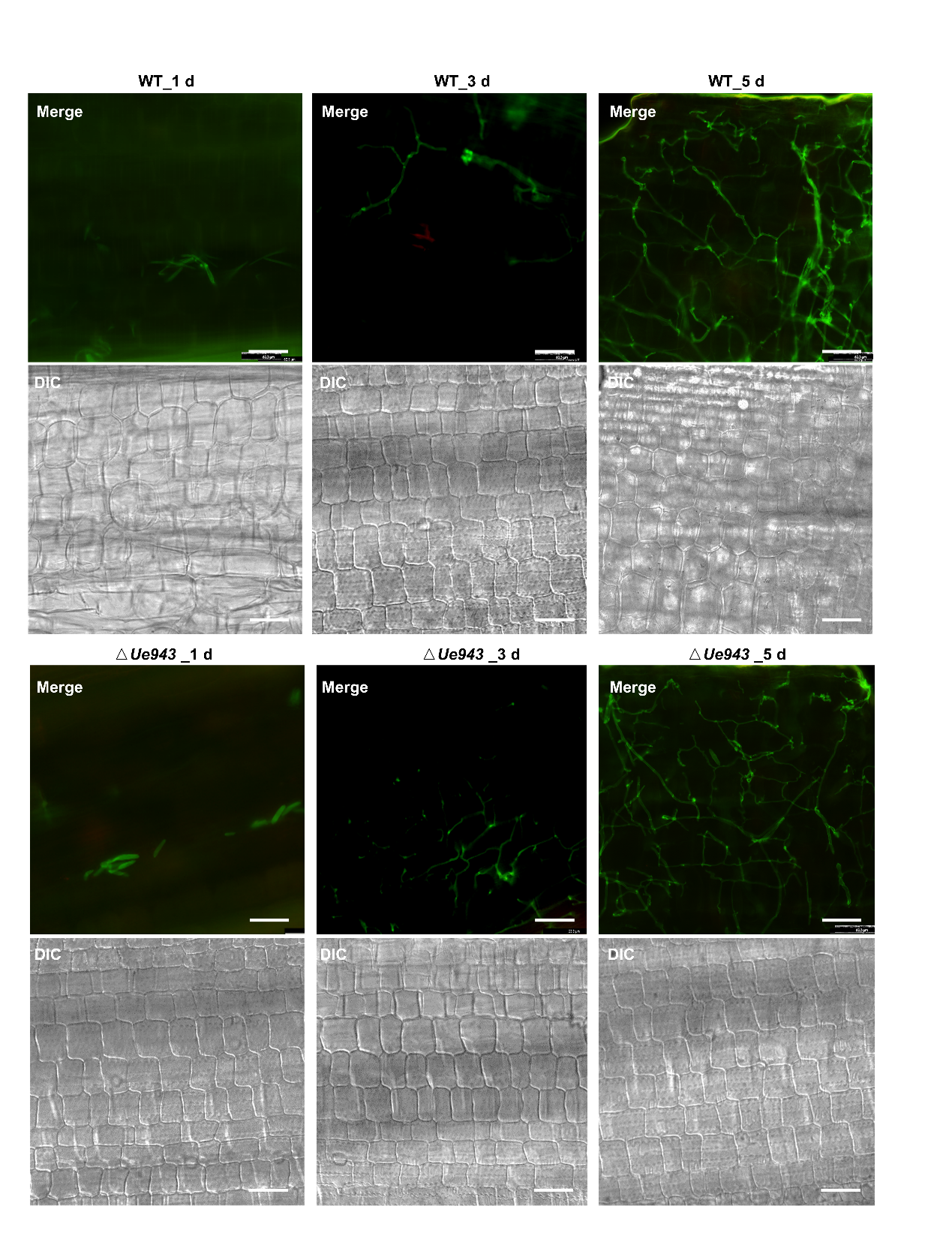


**Figure S3.** Effects of *Ue943* deletion on lignin deposition of plant. The *ΔUe943* strains does not induce a lignin deposition of plant. The mixtures of compatible strains were injected into the stem of *Z. latifolia* and leaf sheaths infected after 1 to 5 d were collected. Fungal hyphae are stained by WGA-AF488 fluorescence (green),lignin deposition is visible by Congo red (red), DIC is used to photograph the structure of plant cells and analyzed by laser scanning confocal microscopy, the scale bars is 62.5 µm.
