# Supplementary material for: The Novel Effector Ue943 Is Essential for Host Plant Colonization by Ustilago esculenta
Source: J Fungi (Basel). 2023 May 19;9(5):593. doi: 10.3390/jof9050593 (PMC10219421; doi:10.3390/jof9050593)
Supplement: Supplementary file 1 [file jof-09-00593-s001.zip › Suppment data/Figure S5.docx]

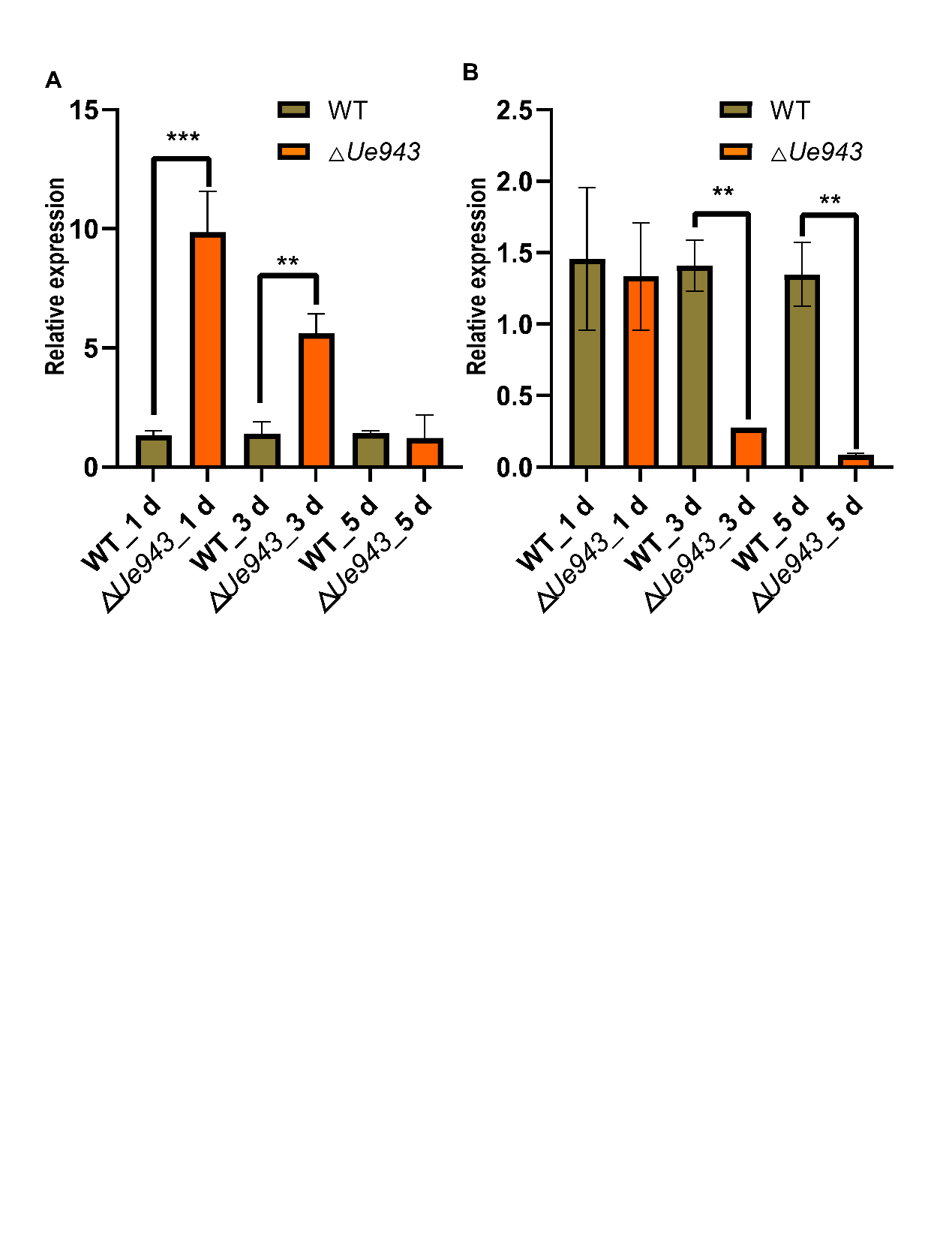


**Figure S5.** Gene expression profile of NADH oxidase and Glucan endo-1,3-beta-glucosidase. Leaf sheaths infected by *U. esculenta* were collected at 1, 3 and 5 days post inoculation and prepared for RT-qPCR. (A) NADH oxidase gene. (B) Glucan endo-1,3-beta-glucosidase gene. Gene relative expression was normalized to the expression of *β-actin.* Mean and standard deviation (SD) were calculated with results from three biological replicates. Significant differences are marked with an asterisk (**, p< 0.01, ***, p< 0.001)
