# Supplementary material for: The Novel Effector Ue943 Is Essential for Host Plant Colonization by Ustilago esculenta
Source: J Fungi (Basel). 2023 May 19;9(5):593. doi: 10.3390/jof9050593 (PMC10219421; doi:10.3390/jof9050593)
Supplement: Supplementary file 1 [file jof-09-00593-s001.zip › Suppment data/Figure S6.docx]

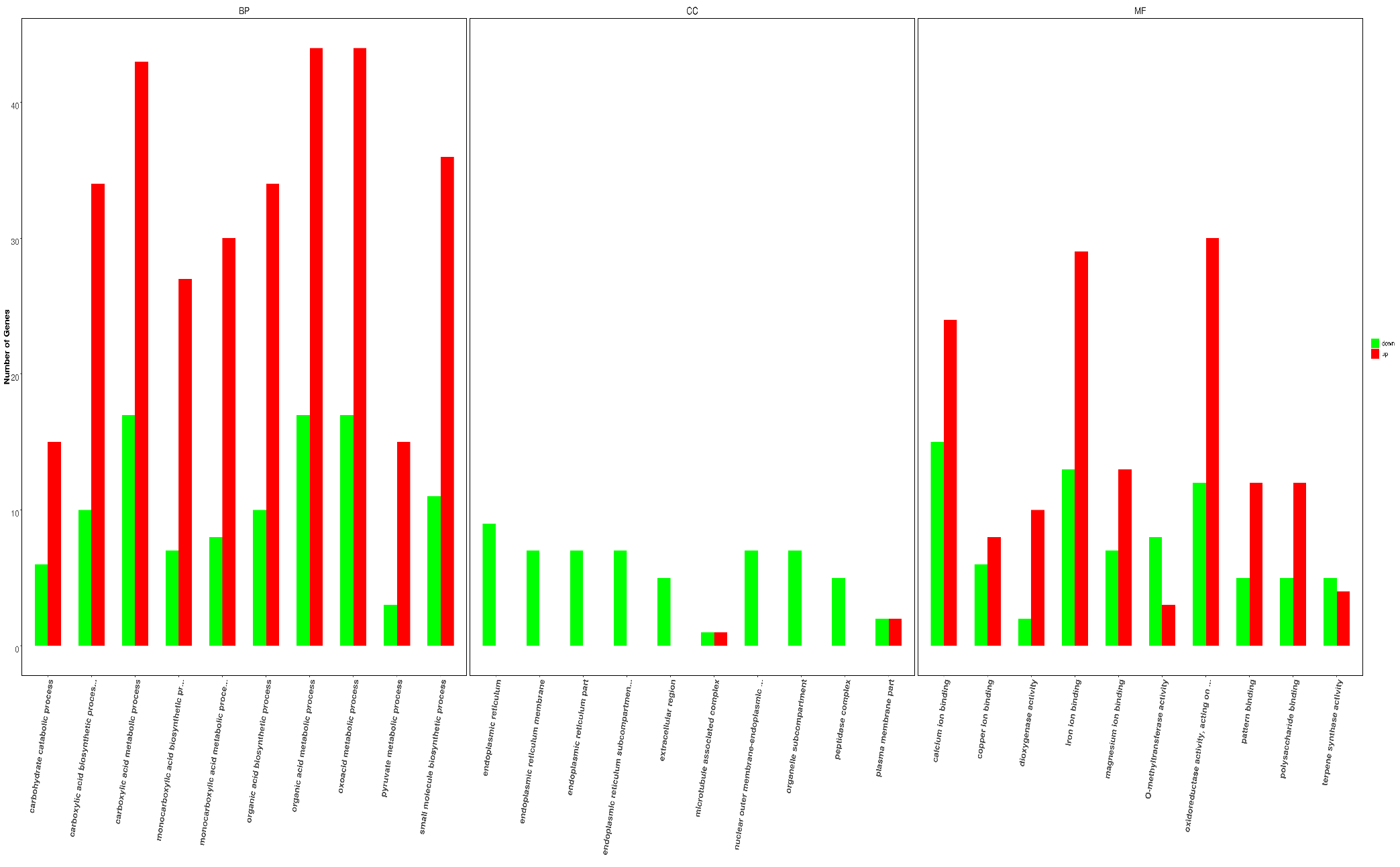


**A**

**DEGs Number of The Most Enriched Go Term**

**△*Ue943*_1 d vs WT_1 d**


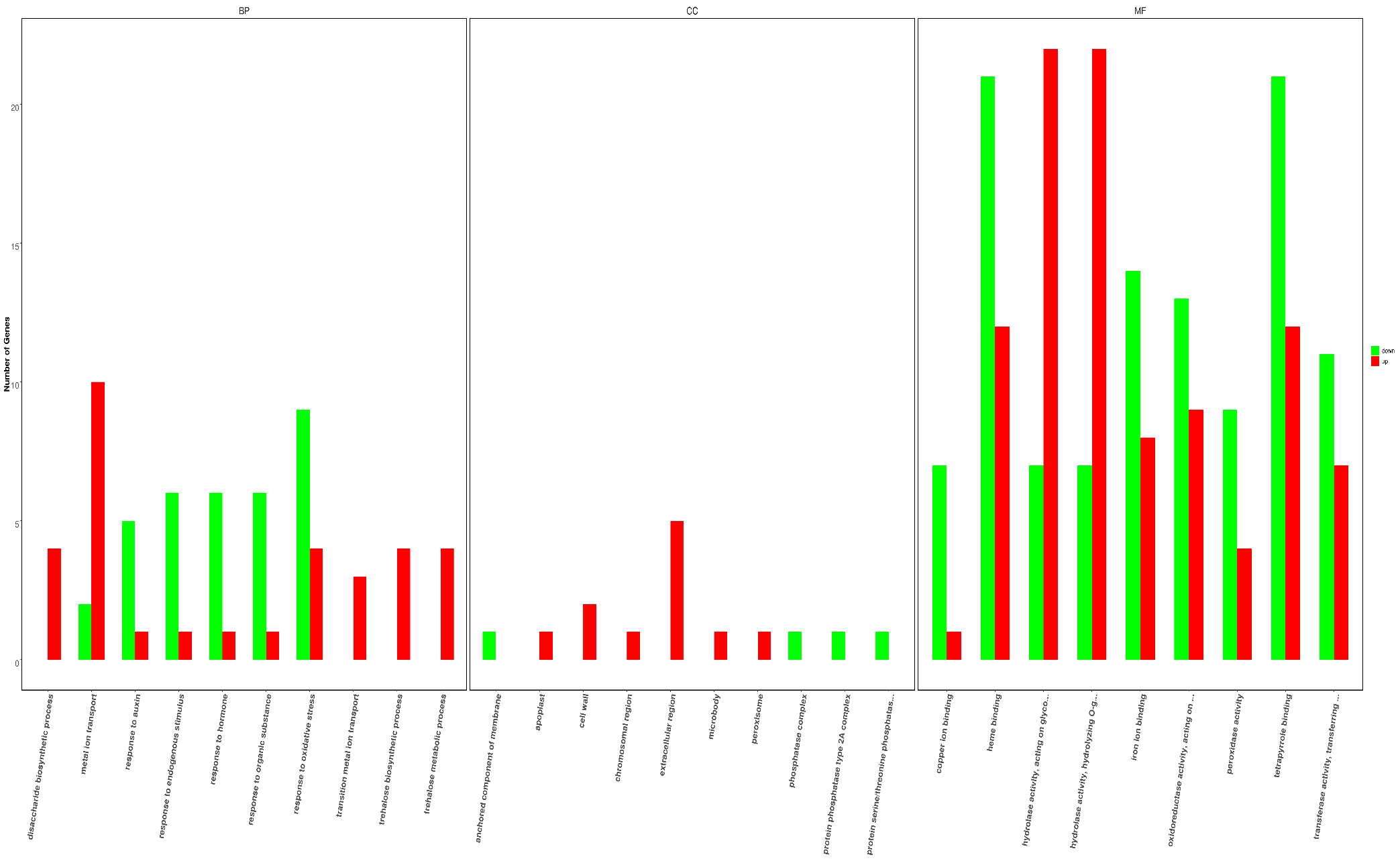


**B**

**DEGs Number of The Most Enriched Go Term**

**△*Ue943*_3 d vs WT_3 d**


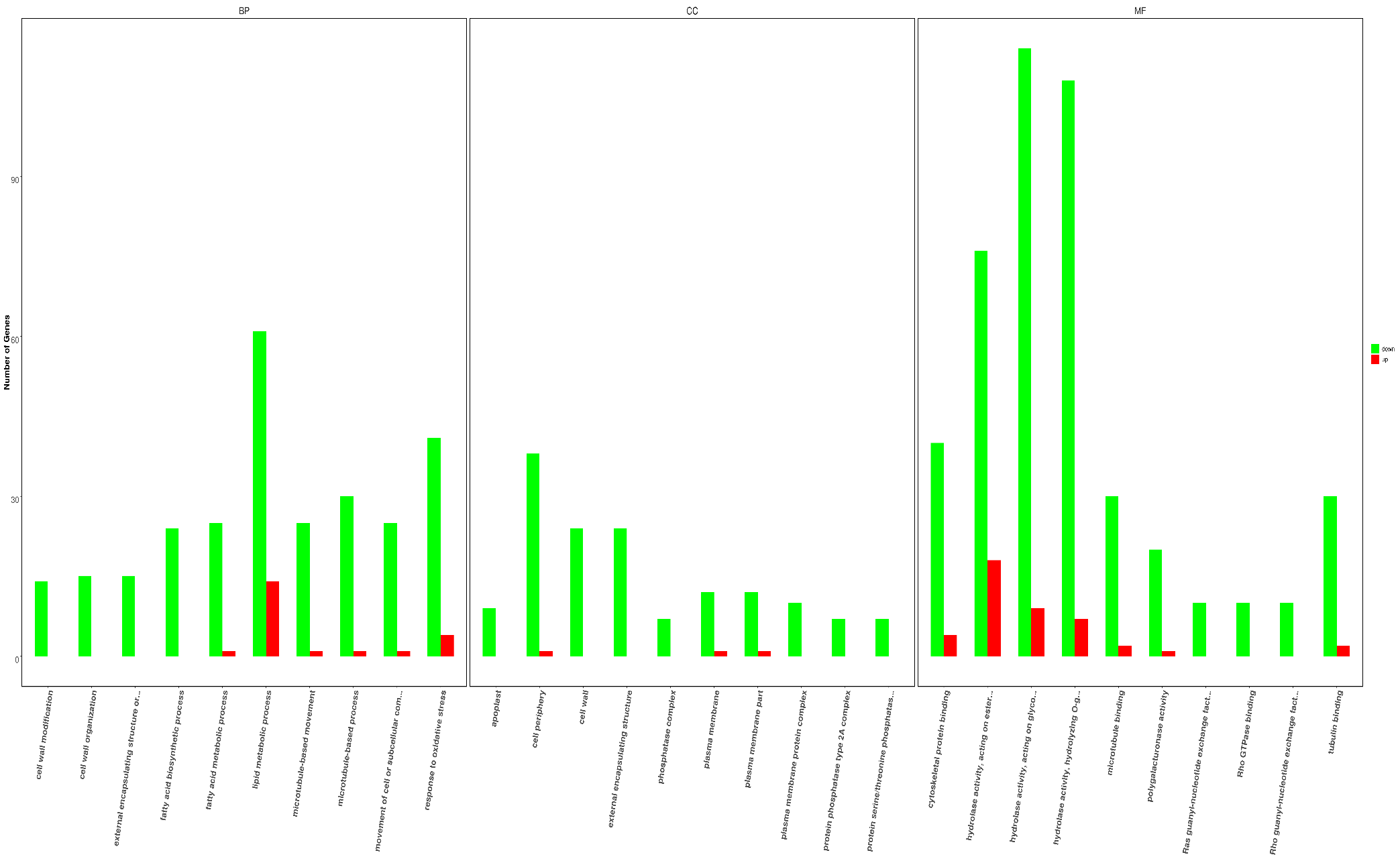


**C**

**DEGs Number of The Most Enriched Go Term**

**△*Ue943*_5 d vs WT_5 d**

**Figure S6**. Enriched Gene Ontology (GO) analysis of upregulated and downregulated gene annotations at 1 d, 3 d and 5 d post inoculation. Gene expression level were compared between host plants infected by WT and Δ*Ue943* strains at 1 d, 3 d, 5 d post inoculation.
