# Supplementary figures and images for: The Novel Effector Ue943 Is Essential for Host Plant Colonization by Ustilago esculenta
Source: J Fungi (Basel). 2023 May 19;9(5):593. doi: 10.3390/jof9050593 (PMC10219421; doi:10.3390/jof9050593)

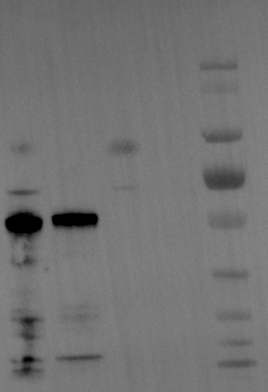

Supplement: Supplementary file 1 [file jof-09-00593-s001.zip › Suppment data/Figure S2.tif]
